# Condorcet Cycles, Contextuality, and the Emergence of Quantum-like Collective States

**DOI:** 10.3390/e28101076

**Published:** 2026-09-30

**Authors:** Leopoldo Trieste, Giuseppe Turchetti

**Affiliations:** Institute of Management, Sant’Anna School of Advanced Studies, 56127 Pisa, Italy

**Keywords:** Condorcet paradox, contextuality, quantum-like models, collective decision-making, emergence, classical representability

## Abstract

The Condorcet paradox is traditionally viewed as a macro-level phenomenon of collective decision-making, showing that individually rational agents with coherent and transitive preferences may nevertheless generate socially intransitive majority outcomes. This interpretation assumes that cyclicity emerges exclusively through aggregation. In this study, we distinguish between micro-Condorcet dynamics, arising from contextual interactions within individual decision processes, and macro-Condorcet dynamics, emerging through majority aggregation. To investigate the relationship between these two levels, we introduce a minimal contextual model in which pairwise decisions are generated sequentially and linked through a contextual coupling mechanism. Although individual agents remain locally coherent and entirely classical at the microscopic level, each decision modifies the context of subsequent comparisons. Simulation results show that increasing contextuality progressively increases the probability of both micro- and macro-level Condorcet cycles. The transition between the two levels is strongly influenced by heterogeneity in decision thresholds, particularly those governing intermediate and closing comparisons. Rather than being driven primarily by initial preference relations, collective cycles emerge from heterogeneous evaluations of intermediate alternatives, which amplify contextual effects and facilitate cycle closure. A critical contextuality threshold is observed beyond which classical representability breaks down and no probability distribution over transitive preference rankings can reproduce the observed pairwise preference probabilities. These findings suggest that quantum-like probabilistic structures may emerge naturally as higher-level representations of contextual collective decision systems.

## 1. Introduction

### 1.1. Local and Global Coherence of Preferences

The distinction between the local and global transitivity of preferences yields four possible configurations. In the case of classical coherence, preferences are transitive at both the individual and collective levels, allowing for a consistent ordering of alternatives throughout the system. Condorcet’s paradox arises when individual transitive preferences generate a collectively intransitive ordering, showing that local coherence does not necessarily imply global coherence. Conversely, contextuality cancellation occurs when individual preferences are locally intransitive, yet aggregation produces a transitive collective ranking. Finally, reinforced contextuality emerges when non-transitivity is present at both levels, so that local contextual effects are preserved or amplified in the collective preference structure. Together, these four cases highlight that coherence and contextuality are not necessarily preserved across levels of analysis, and that the relationship between individual and collective rationality can be considerably more complex than commonly assumed.

Introducing contextuality into preference theory reveals, however, two distinct forms of Condorcet’s paradox. At the micro level, the paradox emerges when context-dependent judgments cannot be reconciled within a single coherent representation of an individual’s preferences. This is an internalized version of Condorcet’s paradox. In the classical formulation, cyclic collective preferences emerge from the aggregation of transitive rankings across different individuals. Here, the same phenomenon arises through the aggregation of judgments generated by a single individual across different contexts. The source of inconsistency is therefore shifted from interpersonal heterogeneity to contextual dependence within the decision-making process itself. In this sense, the individual becomes a population of contexts.

At the macro level, the paradox emerges when coherent individual preferences fail to generate a coherent collective ordering. The first form concerns the relation between contexts and individuals, whereas the second concerns the relation between individuals and society. In both cases, the essential feature is the same: local coherence does not guarantee global coherence. At the macro level, each individual can be viewed as a context on collective preferences. Thus, the two forms of Condorcet’s paradox differ only in the level at which representability breaks down. In the micro form, contexts fail to generate a coherent individual preference structure; in the macro form, individuals fail to generate a coherent collective preference structure. Both cases illustrate the same fundamental principle: local representability does not necessarily imply global representability.

However, a fundamental distinction between micro and macro exists. Non transitivity of local preferences does not need further explanation beyond contextuality. Macro Condorcet’s paradox needs a common rule or a voting rule that forces local behavior to be aggregated into a social order of preferences. Therefore, contextuality explains why a global order may fail to exist locally. Condorcet’s paradox explains why a global order may fail to exist after aggregation.

Under this view, majority voting becomes analogous to a coarse-graining operator in statistical physics.

At the microscopic scale, we have many local preference structures. The voting rule compresses this information into a macroscopic social variable. Condorcet cycles then become an emergent phenomenon, not directly present in any individual voter.

In the micro-Condorcet paradox, incompatibility is cognitive and originates from contextual interactions within the decision maker. In the macro-Condorcet paradox, incompatibility is institutional and originates from the aggregation rule itself. Majority rule generates a collective constraint that is not reducible to any individual preference ordering. As a consequence, collective outcomes exhibit a form of global correlation among pairwise social preferences that is absent at the local level. This situation is reminiscent of quantum correlations, which are not embedded in space-time as local properties of individual particles, but emerge from the structure of the system as a whole.

The majority rule generates a form of institutional correlation among pairwise social preferences. Such correlations do not belong to the space of individual preferences, just as quantum correlations do not belong to space-time viewed as a collection of local properties. In both cases, the relevant structure exists at the level of relations and becomes observable only through its global effects.

### 1.2. Condorcet’s Paradox at a Micro-Level

At a micro-level, Condorcet’s paradox emerges as the effect of contextuality on the capability of a coherent representation of singular preferences.

By the continuity axiom, the utility of alternative *u*(*A*) can be transformed into a probability measure *m* of A in terms m(A)∈[0,1]. For instance, m(A) is the probability that makes indifferent a subject between [1], A for sure, and a mixture of alternatives, the most H and the least B preferred alternatives.

A∼m(A)+(1−m(A)B

Since utility is represented by the probability measure m, an individual Condorcet cycle is:

A≻B, B≻C,C≻A

which implies:

m(A)>m(B), m(B)>m(C),m(C)>m(A),

which leads to the contradiction:

m(A)>m(B)>m(C)>m(A).

Therefore, no global probability measure m  can represent cyclic preferences. Any representation of such preferences must be context dependent.

Therefore, utility cannot be interpreted as an intrinsic property of the alternatives. Instead, utility must be defined relative to the context of comparison. This shift from global to contextual utility representations is the fundamental motivation for a quantum-like approach to decision-making.

### 1.3. Condorcet’s Paradox at a Macro-Level

The traditional significance of Condorcet’s paradox [2] extends far beyond the observation that majority rule may generate cyclical collective preferences. By demonstrating that individually rational and transitive preference orderings can produce an intransitive social ranking, Condorcet (1785) revealed a fundamental instability in collective decision-making. This insight became the starting point for modern social choice theory and directly influenced Arrow’s impossibility theorem [3], which generalized Condorcet’s result by proving that no preference aggregation rule can simultaneously satisfy a set of seemingly reasonable democratic conditions while guaranteeing a coherent social ordering. Subsequently, Sen (1970) [3] expanded the analysis beyond voting procedures, showing through the Liberal paradox that even minimal individual rights may conflict with Pareto efficiency and collective rationality. Together, the contributions [2,3,4] established that the aggregation of individual preferences is subject to deep structural limitations, making conflicts between consistency, efficiency, fairness, and individual liberty an intrinsic feature of collective choice rather than a mere defect of particular voting systems.

The Condorcet paradox has also long been regarded as one of the most striking examples of emergence in collective decision-making. Individually rational agents, each endowed with coherent and transitive preferences, may nevertheless generate aggregate outcomes that are globally inconsistent. The paradox reveals a fundamental tension between local coherence and collective organization, suggesting that the properties of a social system cannot always be reduced to the properties of its constituent individuals.

Classical approaches generally interpret this phenomenon as a consequence of preference aggregation. However, recent developments in complexity theory and quantum cognition have raised a broader question: whether collective systems may undergo transitions that make classical representations progressively inadequate. From this perspective, the central issue is not the existence of cycles themselves, but the conditions under which collective outcomes cease to admit a globally consistent description.

Recent research on the Condorcet paradox has progressively shifted from viewing it as a simple anomaly of preference aggregation to considering it a broader manifestation of collective complexity. Empirical and simulation studies indicate that majority cycles are not merely rare pathological cases, but may arise with significant frequency under realistic preference distributions, renewing interest in the structural origins of collective intransitivity [4,5,6,7].

More recently, the paradox has attracted attention beyond traditional social choice theory. Geometric and topological approaches have interpreted preference cycles as manifestations of global organizational constraints that cannot be reduced to individual preferences. In particular, the inconsistency underlying Condorcet cycles can be associated with non-orientable topological structures such as the Klein bottle and the real projective plane, linking the paradox to broader questions of topology, complexity, and collective organization [8,9].

At the same time, developments in quantum cognition and contextual probabilistic modeling have encouraged a reinterpretation of Condorcet cycles as indicators of collective states that may not admit a classical representation based on mixtures of transitive rankings. Within this broader perspective, contextuality [7] and quantum-like correlations may emerge from the dynamic interaction of self-produced signs and locally coherent decision processes, without requiring pre-existing latent preference structures. Their framework highlights how globally incoherent states can arise from locally consistent interactions, providing a natural link between contextuality, emergence, and non-classical collective behavior [8].

Therefore, whereas quantum effects in physics emerge at extremely small scales, in the social and organizational world, they can be understood as arising from the synthesis of multiple local experiences into a coherent global reality.

An important aspect of the Condorcet paradox is the independence of individual preferences: each voter’s ranking is determined without direct influence from the rankings of other voters. This property is crucial for the quantum formalism, as it reflects the non-signaling condition [7], according to which local choices cannot be used to transmit information and thereby alter the outcomes of other agents. Thus, any collective effects arise from the aggregation mechanism itself rather than from inter-agent signaling.

Consequently, the Condorcet paradox is increasingly viewed not merely as a voting anomaly, but as a potential signature of contextual collective organization and the breakdown of classical latent-order descriptions. This growing convergence between social choice theory, complexity science, topology [10], and quantum-inspired approaches motivates the search for mechanisms through which contextual interactions among classical agents may generate collective structures that are more naturally described by non-classical probabilistic frameworks.

In our view, at the micro level, preferences are not assumed to be fixed and pre-existing. Instead, they are constructed during the decision process, and their expression depends on the sequence of contexts experienced by the decision maker. Different orders of pairwise comparisons may therefore lead to different preference structures. In this sense, contextuality is an intrinsic property of individual preference formation.

At the macro level, however, the situation is different. Individual agents may ultimately express coherent and transitive rankings, either because contextual effects are weak or because a transitive closure mechanism is imposed after the preference construction process. The source of contextuality is then no longer located within a single individual, but in the population as a whole.

Specifically, different individuals may encounter different sequences of pairwise comparisons. For example, one group may evaluate the alternatives according to the sequence AB→BC, a second group according to BC→CA, and a third group according to CA→AB.

Although each individual eventually produces a coherent ranking, the rankings generated by these different contextual histories need not coincide. The population therefore contains heterogeneous but internally consistent preference orderings.

From this perspective, a Condorcet cycle emerges because different groups of individuals experience different orders of contextual realization. The cycle is not produced by irrational agents, nor by individual preference inconsistency, but by the aggregation of rankings that have been constructed under distinct contextual paths. The majority relation can therefore satisfy:

A≻B,B≻C,C≻A,

even when every individual ranking remains transitive.

Consequently, the classical Condorcet paradox may be reinterpreted as a form of *distributed contextuality*. At the individual level, contextuality acts through the sequential construction of preferences. At the collective level, contextuality manifests itself as a distribution of different contextual histories across the population. The Condorcet cycle then appears as an emergent social phenomenon generated by the aggregation of preferences formed under different orders of contextual exposure.

In this interpretation, the fundamental explanatory variable is not the preference itself, but the sequence of contexts through which the preference is constructed. Social cycles arise because individuals do not share the same contextual trajectory, even though each of them may remain perfectly rational and internally consistent.

### 1.4. Aims and Scope

The present study explores this possibility through a minimal contextual model (the model is termed *minimal* because each individual agent is characterized by only one cognitive state and one contextual updating parameter). The objective is to determine the following:

*Micro-Condorcet.* The focus is on the emergence of contextuality at the individual level. Preferences are not assumed to be fixed and pre-existing but are dynamically constructed through sequential pairwise comparisons. Each decision updates the agent’s internal cognitive state through a contextual coupling mechanism governed by the parameter γ. Consequently, subsequent comparisons are evaluated in a modified context generated by previous choices. The model investigates how the strength of contextual coupling affects preference formation and whether sufficiently strong contextual feedback can generate non-classical preference structures, including contextual dependence and preference cycles.

*Macro-Condorcet.* The focus is on the collective consequences of contextual preference formation under majority aggregation. At the population level, two distinct sources of contextuality are present. First, agents may differ in the strength of contextual coupling, represented by the parameter γ. Second, agents may experience different orders of contextual realization, corresponding to different sequences of pairwise comparisons. Thus, individuals are exposed not only to different levels of contextual influence, but also to different contextual trajectories. Even when each agent ultimately produces a coherent ranking, distinct contextual paths may generate different preference orderings across the population. The model therefore investigates how the interaction between contextual updating and heterogeneous comparison sequences shapes collective outcomes under majority rule. In this perspective, Condorcet cycles can be interpreted as an emergent form of distributed contextuality arising from both individual state-dependent preference construction and variations in the order in which contexts are experienced.

### 1.5. Relation to Existing Quantum Approaches to Condorcet Cycles

The interpretation proposed here differs from most existing quantum-inspired approaches to social choice and preference aggregation. In the quantum cognition literature [9,11,12,13], contextuality, superposition, and Hilbert-space representations are typically introduced as alternative probabilistic frameworks capable of describing violations of classical rationality and order effects. In these models, the quantum formalism serves primarily as a representational tool.

The present approach follows a different logic. Rather than assuming a quantum-like structure from the outset, the model begins with locally coherent and entirely classical agents. Contextual interactions are then introduced at the level of the decision process, allowing collective properties to emerge dynamically. It directly elaborates on the perspective developed in [14].

From this perspective, Condorcet cycles constitute the first observable manifestation of a broader transformation in the collective organization of preferences. As contextual interactions increase, the system eventually reaches a regime in which classical representations based on mixtures of transitive rankings cease to exist. At the same time, a low-dimensional quantum-like representation continues to reproduce the observed probabilities with minimal error.

The significance of this result lies in the possibility of deriving the need for a quantum-like description from the internal dynamics of the system itself. Rather than postulating contextuality, the model identifies a mechanism through which contextuality emerges. The quantum formalism appears only after the breakdown of classical representability, suggesting that quantum-like structures may arise as macroscopic properties of collective decision systems.

In this sense, the model proposes a bridge between classical social choice theory and quantum cognition. Condorcet cycles are interpreted not merely as voting paradoxes, but as indicators of a critical transition from globally ordered preference structures to irreducibly contextual collective states. The quantum-like representation then becomes a natural consequence of this transition rather than an initial assumption.

### 1.6. The Contextual Emergence Model

The model begins with a population of agents evaluating three alternatives, A, B, and C. In fact, the Condorcet paradox requires at least three alternatives. With only two alternatives, majority preferences are necessarily transitive and can be represented along a single dimension. The introduction of a third alternative allows the emergence of preference cycles, transforming the structure of collective preferences from a linear ordering into a closed geometric configuration. In this sense, the paradox is intrinsically spatial: it introduces a minimal relational geometry in which collective preferences can be represented as orientations or angles between alternatives rather than as positions along a single axis.

The third alternative plays a role analogous to an additional degree of freedom. With only two alternatives, preferences can be represented along a single dimension, and no Condorcet cycle can emerge. The introduction of a third alternative creates a closed relational structure in which global inconsistencies become possible. In this sense, the third alternative does not act as an observer, but as the minimal ingredient required for the emergence of collective contextual effects. With three alternatives, the final comparison is no longer evaluated in isolation. Its interpretation depends on the relational structure generated by the preceding comparisons.

At the individual level, coherence is preserved throughout the process. No agent is assumed to possess cyclic or irrational preferences. Instead, each decision is generated from an underlying cognitive state and influences the subsequent decision through a contextual coupling parameter γ. When contextual coupling is absent, decisions are effectively independent and collective outcomes can be interpreted as the aggregation of stable latent preferences. As γ increases, however, the outcome of each judgment progressively modifies the state from which subsequent judgments are produced. Preferences cease to behave as static objects and become dynamically generated through the decision process itself. This mechanism introduces contextuality without presupposing any quantum structure. The model remains entirely classical at the microscopic level. Nevertheless, the accumulation of local contextual effects can profoundly alter the behavior of the collective system.

### 1.7. From Local Coherence to Collective Cycles

The first observable manifestation of this transformation is the emergence of Condorcet cycles. While no individual exhibits a cyclic ordering, the collective system increasingly generates the pattern A>B,B>C,C>A.

The importance of this result lies in the fact that the cycle is not introduced at the individual level. It emerges from the network of contextual dependencies connecting successive judgments.

In this sense, the Condorcet cycle functions as an order parameter of the transition. It signals that the global structure of the system is beginning to acquire properties that are absent at the local level.

The quantity K=P(A>B)+P(B>C)+P(C>A)  can measure the phenomenon.

For any transitive preference ordering, at most two of these relations can simultaneously hold. Consequently, any classical mixture of transitive rankings must satisfy K≤2.

This inequality defines a classical consistency bound. The contextuality index Δ=K−2  measures the degree to which the system departs from this classical regime. As long as Δ≤0, the observed probabilities remain compatible with a classical latent-order representation. When Δ becomes positive, the collective dynamics begin to exceed the constraints imposed by globally ordered preference structures.

The emergence of positive values of Δ therefore indicates a qualitative change in the organization of the system rather than a simple quantitative increase in cyclic behavior.

### 1.8. Complexity and Collective Organization

The transition is further characterized through entropy and correlation measures. Entropy provides information about the diversity of configurations explored by the system. Low entropy corresponds to highly ordered behavior, whereas high entropy reflects the coexistence of multiple competing configurations.

The simulations show that entropy tends to reach a maximum near the critical region where contextuality emerges. This behavior is characteristic of many complex systems approaching a phase transition. Near the critical point, the system explores a large number of possible configurations before settling into a new organizational regime.

At the same time, correlations among pairwise comparisons increase substantially. These growing correlations reveal the formation of collective organization. Local decisions cease to behave as independent events and become integrated into a larger structure generated by contextual interactions.

### 1.9. The Loss of Classical Representability

For every value of the contextual coupling parameter, we examined whether the observed probabilities could be reproduced by a non-negative probability distribution over the transitive preference rankings.

At low levels of contextual coupling, such a representation exists. Collective behavior can therefore be interpreted as the outcome of a classical mixture of coherent preference orderings.

Beyond a critical threshold, however, the feasibility test fails. No probability distribution over the six rankings can reproduce the observed pairwise probabilities. The collective state thus loses classical representability.

This result marks the most significant turning point in the model. Individual judgments remain well-defined and internally consistent. What disappears is the possibility of embedding those judgments within a single globally consistent preference structure.

### 1.10. Non-Classical Representation

The failure of classical representability naturally raises the question of whether an alternative representation exists. To address this issue, the observed probabilities were fitted using a minimal quantum-like model based on a low-dimensional state vector and the Born probability rule. Remarkably, the resulting prediction errors remained extremely small, even in the parameter region where classical representations no longer existed.

This finding should be interpreted carefully. It does not imply that decision makers are quantum systems or that physical quantum processes are involved in collective choice. Rather, it indicates that the probabilistic structure generated by contextual interactions can be represented effectively within a quantum-like framework after classical latent-order descriptions break down.

The significance of the quantum model therefore lies in its descriptive power. While the classical representation becomes impossible, the quantum-like representation continues to provide an accurate and compact account of the observed behavior.

## 2. Materials and Methods

### 2.1. Simulation Framework

To investigate the emergence of micro-level Condorcet’s cycles, a minimal agent-based simulation was developed in which individual decisions remain locally coherent while collective structures are allowed to emerge through contextual interactions.

The model considers three alternatives, A, B, and C. Rather than assuming fixed preference rankings, agents generate a sequence of pairwise comparisons whose outcomes depend on an evolving internal cognitive state. This approach allows preferences to be dynamically constructed during the decision process rather than being treated as static latent variables.

Each simulated agent is initialized with a latent cognitive state x∼U(0,1), where U(0,1) denotes the uniform distribution over the interval [0,1]. The variable x represents an initial disposition prior to any decision. The first comparison concerns alternatives A and B. The outcome is generated according to:

(1) $$AB=\{\begin{array}{cc} 1 & if\;x>0.1\\ 0 & otherwise \end{array}$$

After the first decision, the internal state is updated through a contextual coupling mechanism:

(2) $$x_{2}=x+\gamma (AB-0.5),\;$$

where γ∈[0,1]  is a contextual coupling parameter controlling the degree to which previous decisions influence future ones.

The second pairwise comparison is then computed as:

(3) $$BC=\{\begin{array}{cc} 1 & if\;x_{2}>0.5\\ 0 & otherwise \end{array}$$

The state is updated again according to:

(4) $$x_{3}=x_{2}+\gamma (BC-0.5),\;$$

and the final comparison is generated as:

(5) $$CA=\{\begin{array}{cc} 1 & if\;x_{3}>0.8\\ 0 & otherwise \end{array}$$

State variables were constrained to remain within the interval [0,1] after each update.

The parameter γ therefore controls the strength of contextual effects. When γ=0, decisions are effectively independent. Increasing values of γ progressively strengthen the dependence of each judgment on previous judgments, generating contextual interactions without introducing any explicit quantum assumptions.

The model does not assume quantum mechanics. However, it does introduce contextual dependence at the microscopic level through the coupling parameter γ. Consequently, the simulation should not be interpreted as demonstrating the spontaneous emergence of contextuality. Rather, it investigates how local contextual interactions propagate to the collective level and under which conditions they generate macroscopic states that lose classical representability.

### 2.2. Parameter Exploration

The contextual coupling parameter varied over the interval 0 ≤ γ ≤ 1 with increments of 0.02.

For each value of γ, *N* = 10,000 agents were simulated. Collective probabilities were estimated from the resulting population frequencies.

The probability of a complete Condorcet cycle was defined as

(6) $$P_{cycle}=P(AB=1,\;BC=1,\;CA=1).\;\;$$

This quantity was used as an indicator of the emergence of globally cyclic preference structures.

### 2.3. Contextuality Measure

To quantify deviations from classical preference ordering, the quantity

(7) K=P(A>B)+P(B>C)+P(C>A) 

was introduced. For any mixture of transitive rankings, at most two of the three relations can simultaneously hold. Consequently, K≤2 defines a classical consistency bound.

The contextuality index was defined as Δ=K−2. Negative or null values indicate compatibility with a classical preference representation, whereas positive values indicate that the observed probabilities exceed the classical bound.

### 2.4. Classical Feasibility Test

The existence of a classical representation was evaluated by determining whether the observed probabilities could be reproduced by a non-negative probability distribution [14,15,16] over the six transitive rankings ABC, ACB, BAC, BCA, CAB, CBA.

This problem was formulated as a linear feasibility task [13]. Let $p_{1},\ldots ,\;p_{6}$ be the probability distribution assigned to the six rankings. The observed probabilities were represented by:

(8) $$b=\left(\begin{array}{c} P(A>B)\\ P(B>C)\\ P(C>A) \end{array}\right)$$

and feasibility was assessed by Ap=B  subject to $p_{i}>0$, $\sum_{i=1}^{6}p_{i}=1$ [17].

A feasible solution indicates the existence of a classical latent-order representation. Failure to find a feasible solution implies that the collective state cannot be represented as a mixture of transitive rankings.

### 2.5. Quantum-like Representation

To investigate whether an alternative probabilistic representation remained available beyond the loss of classical feasibility, a minimal quantum-like model was fitted to the observed probabilities.

The model employed a two-dimensional state vector:

(9) $$|\psi \rangle =\left(\begin{array}{c} \cos\theta \\ \sin\theta \end{array}\right)\;$$

together with three measurement vectors corresponding to the comparisons A > B, B > C, and C > A. Probabilities were computed according to the Born rule:

(10) $$P\left(X\right)=|\left\langle X|\psi \right\rangle {|}^{2}\;$$

Model parameters were estimated by minimizing the sum of squared errors between the observed and predicted probabilities.

Model performance was assessed using the root mean squared error (RMSE):

(11) $$RMSE=\sqrt{\frac{1}{3}\Sigma_{i}{\left(P_{i}^{obs}-P_{i}^{model}\right)}^{2}}\;,\;$$

allowing for a direct comparison between the observed collective behavior and the quantum-like representation across the entire range of contextual coupling values.

The resulting framework makes it possible to examine whether the emergence of contextual collective states coincides with the breakdown of classical representability and the persistence of a low-dimensional quantum-like description.

Finally, we conducted a sensitivity analysis to examine the probability of observing Condorcet cycles at the micro-level as the model parameters vary.

For each simulation run, values of the contextual coupling parameter γ and the three decision thresholds $\left(t_{AB},t_{BC},t_{CA}\right)$ were sampled from the interval [0,1] using Latin hypercube sampling (LHS) [15]. This procedure generated 1000 parameter configurations that uniformly covered the multidimensional parameter space.

For each parameter configuration, a population of agents was simulated. Agents were initialized with a latent cognitive state, and pairwise comparisons were generated sequentially according to Equations (1)–(5). The resulting individual preference patterns were the proportion of simulations yielding a Condorcet cycle was computed. This proportion provided an estimate of the probability of observing individual preference cyclicity under a given parameter configuration.

### 2.6. Condorcet’s Cycles and Contextuality at a Macro Level

The model was analyzed using a Latin hypercube probability analysis (LHPA) framework to investigate how individual-level contextual decision processes generate collective Condorcet outcomes. For each scenario, a population of N individuals was generated, where every individual was assigned a distinct contextual coupling parameter $\gamma_{i}$ and three decision thresholds $\tau_{AB,i}$, $\tau_{BC,i}$, and $\tau_{CA,i}$. The distributions of these parameters were allowed to vary across scenarios through beta distributions whose shape parameters were sampled using Latin hypercube sampling. Individual decisions were then computed sequentially according to the contextual update model, producing the pairwise outcomes $AB_{i}$, $BC_{i}$, and $CA_{i}$. An individual was classified as exhibiting a *micro-Condorcet cycle* when $AB_{i}=BC_{i}=CA_{i}=1$. At the collective level, majority rule was applied separately to each pairwise comparison to obtain the macro outcomes $AB_{M}$, $BC_{M}$, and $CA_{M}$, from which the presence or absence of a *macro-Condorcet cycle* was determined.

To study the micro-to-macro transition, four mutually exclusive probabilities were defined: CC (macro Condorcet given micro Condorcet), CNC (macro non-Condorcet given micro Condorcet), NCC (macro Condorcet given micro non-Condorcet), and NCNC (macro non-Condorcet given micro non-Condorcet). These quantities capture the persistence, disappearance, emergence, and absence of Condorcet cycles across levels of aggregation. The resulting probabilities were analyzed as functions of the mean and standard deviation of γ, $\tau_{AB}$, $\tau_{BC}$, and $\tau_{CA}$, allowing the identification of parameter regions in which contextuality at the individual level is amplified, suppressed, or transformed by majority aggregation into collective cyclic behavior (see Table 1). This approach provides a quantitative characterization of the conditions under which micro-level contextual dynamics propagate to, or are modified by, macro-level social choice outcomes.

## 3. Results

### 3.1. Condorcet’s Cycles at a Micro-Level

**Figure 1:**
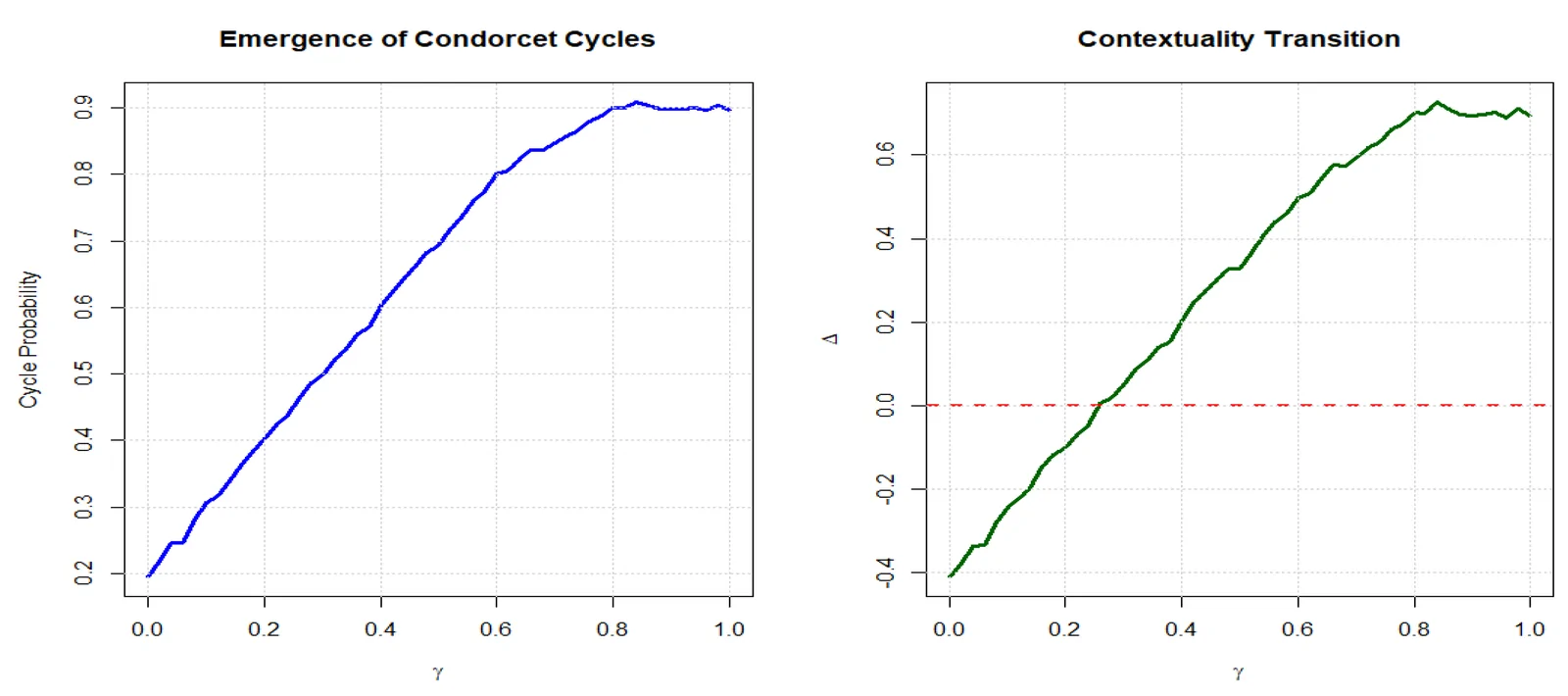
Probability of emergence of Condorcet cycles and the contextuality transition as γ increases. For γ>0, the model exhibits increasingly contextual behavior.

The left panel of Figure 1 shows that the probability of observing a Condorcet cycle increases steadily as the contextual coupling parameter γ increases. At low levels of contextual interaction, cyclic collective preferences are relatively uncommon, indicating that local decisions remain only weakly coupled. As contextual influence strengthens, the probability of generating the cycle A>B, B>C, C>A rises almost monotonically until it approaches a saturation regime.

This result suggests that Condorcet cycles are not isolated anomalies, but collective structures generated by contextual interactions among locally coherent decisions. The increasing frequency of cycles indicates that contextual dependencies progressively reorganize the decision space, favoring globally intransitive outcomes despite the preservation of local consistency. In this sense, the Condorcet cycle emerges as a macroscopic property of the system and may be interpreted as the first observable signature of collective self-organization.

The right panel of Figure 1 displays the contextuality index Δ = K−2. The horizontal dashed line at Δ= 0 represents the classical consistency boundary. Values below this threshold are compatible with a classical representation based on mixtures of transitive preference rankings.

The figure reveals a clear transition. For small values of γ, the contextuality index remains negative, indicating that the observed probabilities can still be interpreted within a classical framework. As contextual coupling increases, Δ progressively rises and crosses zero around $\gamma_{c}\approx 0.25$.

Beyond this critical region, the system enters a contextual regime in which the observed probabilities exceed the classical consistency bound.

The significance of this crossing is that it marks the onset of collective structures that cannot be straightforwardly reduced to a globally ordered preference system. Local decisions remain well-defined, but their aggregate organization begins to display properties that are incompatible with classical latent-order representations.

**Figure 2:**
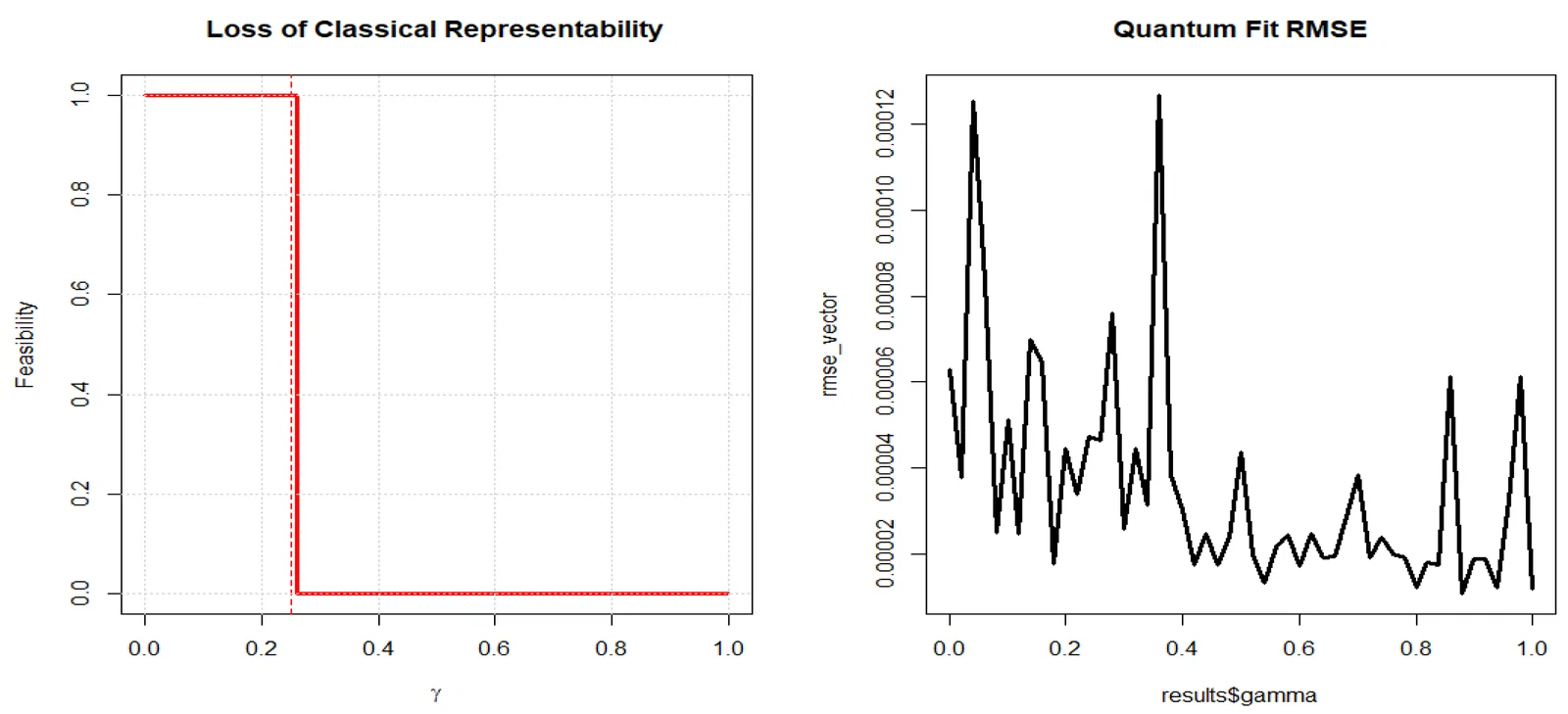
Breakdown of classical representability and quantum-like model accuracy.

The left picture of Figure 2 reports the outcome of the classical feasibility analysis. The binary nature of the curve reflects whether the observed probabilities can be reproduced by a non-negative probability distribution over the six transitive rankings. A value of one indicates that a classical representation exists, whereas a value of zero indicates that no such representation can be found.

For values below $\gamma_{c}\approx 0.25$, the collective probabilities remain compatible with a classical latent-order model. Although contextual interactions are already present, the resulting behavior can still be interpreted as a mixture of globally transitive preference orderings. The collective state therefore remains reducible to classical preference structures.

Beyond the critical threshold, feasibility collapses abruptly to zero. At this point, no probability distribution over the six transitive rankings is capable of reproducing the observed pairwise probabilities. The collective system therefore loses classical representability. Importantly, this transition does not arise from a loss of individual rationality. Local decisions remain governed by the same underlying rules, while the collective organization becomes incompatible with any globally consistent ordering.

The abrupt nature of the transition suggests that the system undergoes a qualitative reorganization rather than a gradual quantitative change. The critical value $\gamma_{c}$ thus marks the boundary between a classically representable regime and an emergent contextual regime.

The right panel of Figure 2 reports the root mean squared error (RMSE) of the quantum-like representation. The error remains extremely small across the entire parameter range, typically on the order of $10^{-4}$, because of the global nature of the Condorcet paradox with respect to local transitivity.

This indicates that the quantum-like model reproduces the observed probabilities with very high accuracy.

The most significant feature of the figure is that the prediction error remains low even after the disappearance of classical feasibility. In other words, the breakdown of the classical representation is not accompanied by a loss of probabilistic structure. Although the collective state can no longer be represented as a mixture of transitive rankings, it continues to admit an accurate low-dimensional quantum-like description.

The stability of the RMSE across the transition suggests that the emergent contextual regime remains highly organized. Rather than becoming random or unstructured, the collective dynamics appear to reorganize into a form that is more naturally captured by a quantum-like probabilistic framework.

Taken together, the two figures reveal the central result of the study. As contextual coupling increases, the system reaches a critical threshold at which classical representability of singular preferences disappears. However, this loss of classical structure does not imply a breakdown of predictability. On the contrary, the quantum-like model continues to reproduce the observed probabilities with negligible error.

The transition may therefore be interpreted as a shift from a regime in which the collective behavior of singular agents can be represented through classical preference orderings to a regime in which such representations become impossible, while a quantum-like representation remains valid, thus marking the emergence of a general state that is irreducibly contextual yet still highly structured.

**Figure 3:**
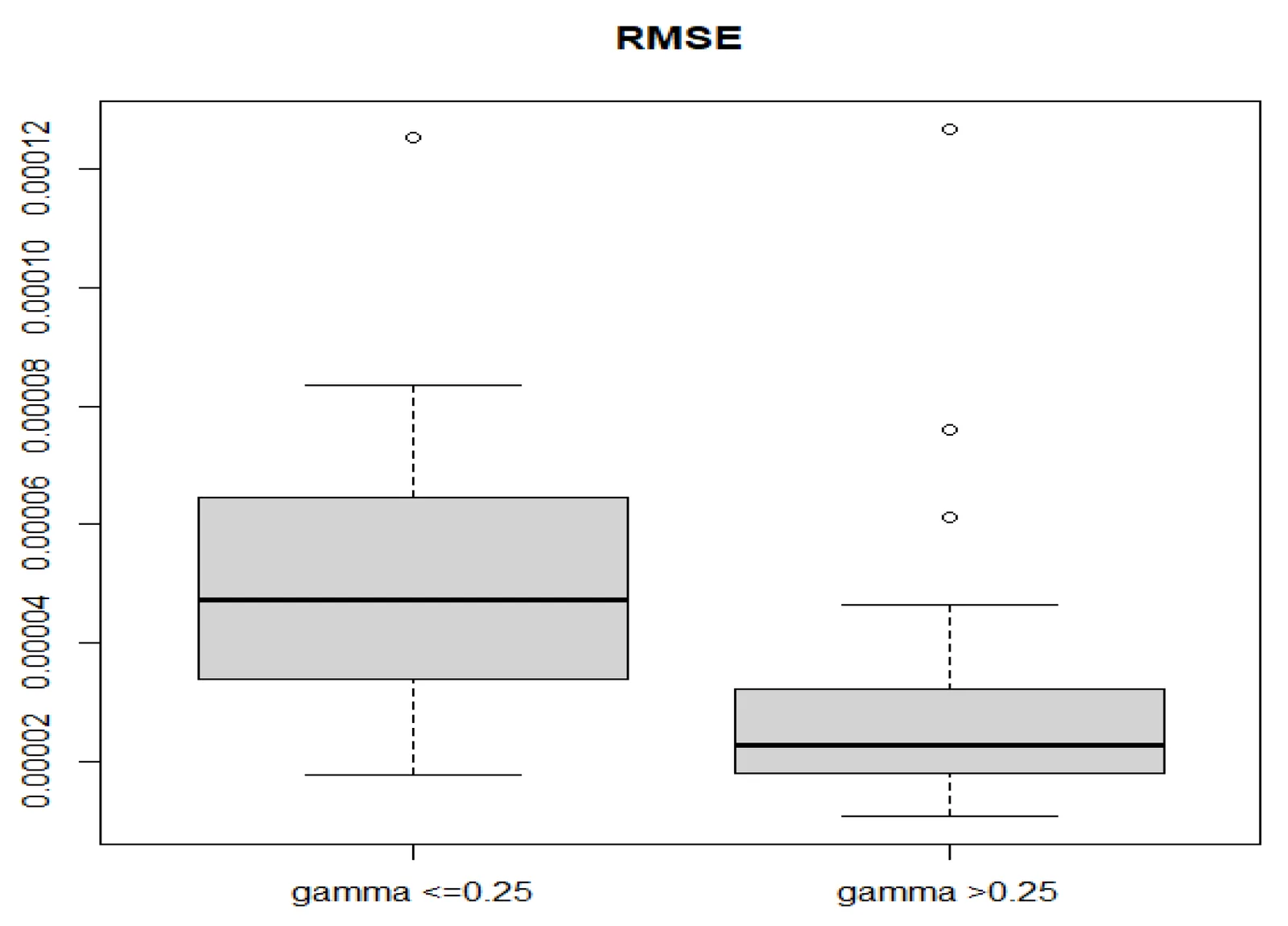
Comparison of RMSE distributions with γ≤0.25, γ>0.25.

Figure 3 compares the distribution of quantum-model prediction errors in the pre-critical (γ ≤ 0.25) and post-critical (γ > 0.25) regimes.

A visible downward shift in the distribution was observed after the critical threshold. The median error, the interquartile range, and the overall spread of the distribution all decreased in the post-critical regime, indicating a more accurate fit of the quantum-like model after the onset of contextuality. Although a few outliers remained present, the central tendency of the error distribution was consistently lower beyond the transition.

This visual pattern is supported by a Welch two-sample *t*-test, which revealed a significant difference between the two regimes (t =2.63, p=0.018). The mean prediction error decreased from $5.29\times 10^{-5}\;$ before the transition to $2.96\times 10^{-5}$ after the transition.

The result is particularly noteworthy because it occurs precisely where classical feasibility disappears. As the contextual coupling increases and the collective state becomes incompatible with any mixture of transitive rankings, the quantum-like representation does not deteriorate. On the contrary, its predictive accuracy improves slightly.

These findings suggest that the emergent contextual regime is not characterized by increased randomness or reduced predictability. Instead, the collective system appears to reorganize into a structure that is less compatible with classical preference representations while remaining highly consistent with a quantum-like probabilistic description.

### 3.2. Analysis of the Thresholds

The thresholds were initially fixed at 0.3, 0.5, and 0.8 to illustrate an example of cyclical preference induced by contextuality. However, a more informative analysis involves examining the sensitivity of the system as these thresholds vary across the interval [0,1].

**Figure 4:**
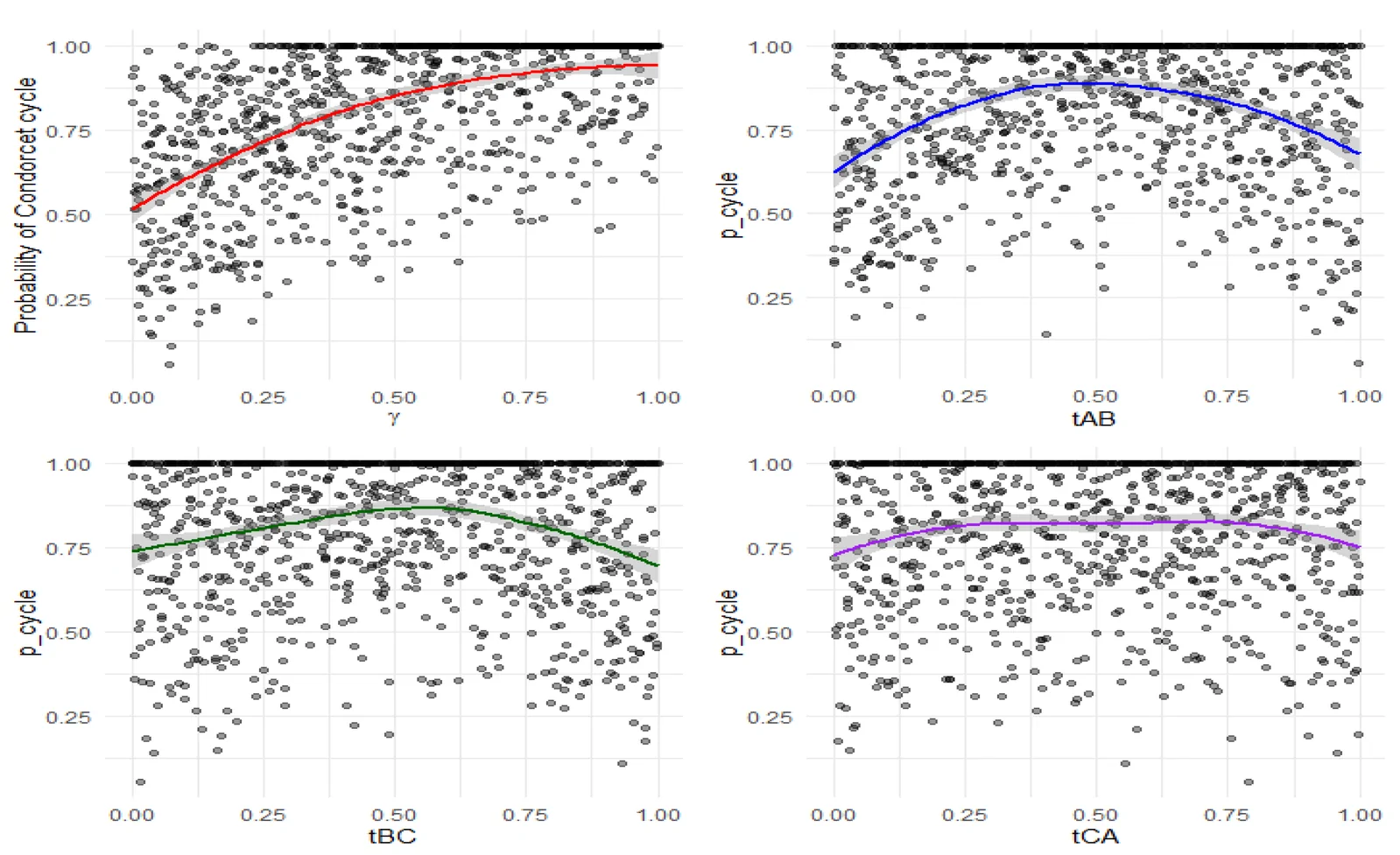
Effect of contextual coupling and decision thresholds on the probability of micro-Condorcet cycles.

Figure 4 summarizes the results of a Monte Carlo simulation of the contextual decision-making model adopting a probability sensitivity analysis.

Each point in the figure represents one parameter configuration obtained through LHS. The corresponding colored curves indicate the average relationship between each parameter and the probability of observing a Condorcet cycle.

The upper-left panel shows a strong positive relationship between the contextual coupling parameter and the probability of cyclic preferences. As γ increases, previous decisions exert a stronger influence on subsequent evaluations, progressively amplifying sequential dependencies. Consequently, the probability of observing a Condorcet cycle increases monotonically, reaching values close to one for large values of γ. This result suggests that contextual feedback is the primary mechanism generating intransitive preference structures in the model.

The upper-right panel reveals a nonlinear, inverted U-shaped relationship between the first decision threshold and the probability of cycles. Intermediate values of $t_{AB}$ are associated with the highest levels of cyclicity, whereas very low or very high thresholds reduce the occurrence of cycles. When the threshold is extreme, the first comparison becomes almost deterministic, leaving less variability for contextual amplification to operate.

A similar pattern emerges for the second threshold $t_{BC}\;$ (lower-left panel). The probability of observing a cycle is maximized for intermediate threshold values and decreases toward both ends of the parameter range. Because the BC comparison occupies an intermediate position in the decision sequence, a moderate level of uncertainty appears to facilitate the propagation of contextual effects across successive choices.

The lower-right panel indicates that the final threshold also exhibits a weak inverted U-shaped relationship with cycle probability. Although the effect is less pronounced than for γ, intermediate threshold values again seem to favor the emergence of cyclic preferences. Extremely permissive or restrictive thresholds reduce the opportunity for the cumulative contextual dynamics to manifest as observable preference cycles.

The emergence of micro-Condorcet cycles is driven primarily by the strength of contextual coupling, while the three decision thresholds play a secondary but non-negligible role. Cyclic preferences are most likely to arise when (i) contextual influence is strong and (ii) decision thresholds remain at intermediate levels, thereby preserving sufficient variability in each pairwise comparison. These findings suggest that violations of transitivity can emerge naturally from the interaction between sequential contextual updating and probabilistic decision thresholds, even when agents are characterized by a single underlying latent state.

### 3.3. From Micro to Macro

**Figure 5:**
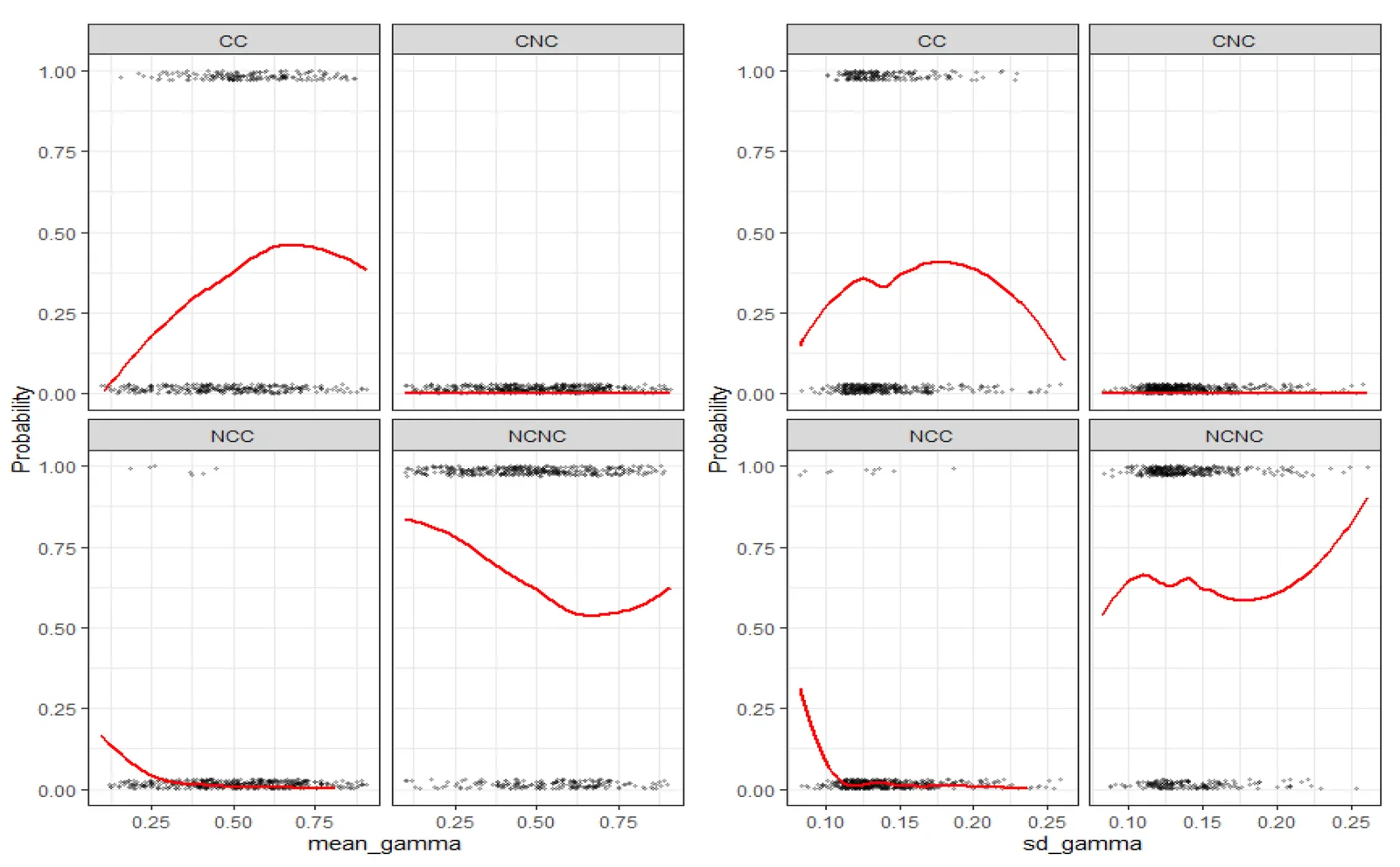
Latin hypercube probability analysis (LHPA) of micro-to-macro Condorcet’s paradox (as function of γ). CC (macro-Condorcet given micro-Condorcet), CNC (macro-non-Condorcet given micro-Condorcet), NCC (macro-Condorcet given micro-non-Condorcet), and NCNC (macro-non-Condorcet given micro-non-Condorcet).

Figure 5 shows the relationships between the first two moments (mean and standard deviation) of the contextuality parameter γ and the four possible micro-to-macro transitions: CC, CNC, NCC, and NCNC. The results reveal how both average contextuality and population heterogeneity shape the propagation of Condorcet cycles from the individual to the collective level. The mean parameters describe the average tendency of the population toward contextual cyclic behavior, whereas the standard deviations quantify how population heterogeneity amplifies, suppresses, or transforms the transmission of Condorcet cycles from the micro to the macro level.

*Mean* γ. The probability that a micro-level cycle persists at the macro level increases with mean contextual coupling, reaching a maximum at intermediate-high values of γ.

The suppression of a micro-level cycle by majority aggregation remains essentially negligible across the entire range of mean γ.

The emergence of a macro-level cycle from predominantly non-cyclic individuals rapidly declines as mean γ increases. Non-cyclic states tend to remain non-cyclic at the macro level, although this persistence weakens slightly at intermediate values of γ.

SD γ. Moderate heterogeneity in contextual coupling supports cycle persistence, whereas high heterogeneity reduces the transmission of cycles to the macro level. Variation in γ has almost no effect on the probability that a micro-level cycle disappears after aggregation. Macro-level cycles rarely emerge from non-cyclic micro populations, and this probability approaches zero as heterogeneity increases. Greater heterogeneity in γ strengthens the stability of non-cyclic collective outcomes.

Notably, the NCC transition corresponds to the classical Condorcet paradox: when contextuality is weak, a collective Condorcet cycle can emerge despite the absence of cycles at the individual level. Thus, the majority rule itself acts as the source of cyclicity. As contextual coupling increases, this purely emergent macro-level effect progressively disappears, and collective cycles become increasingly tied to cycles already present at the micro level.

The most notable result concerns the heterogeneity of the BC and CA decision thresholds. While changes in the mean threshold values mainly affect the persistence or disappearance of existing cycles, increasing variability in $\tau_{BC}$ and especially $\tau_{CA}$ substantially raises the probability of observing a macro-level Condorcet paradox. In particular, higher values of $SD\left(\tau_{CA}\right)$ are associated with an increase in both the persistence of micro-level cycles (CC) and the emergence of macro cycles from predominantly non-cyclic micro states (NCC), accompanied by a reduction in non-cyclic outcomes (NCNC). These findings suggest that population heterogeneity, rather than average decision tendencies alone, plays a crucial role in generating collective intransitivities through majority aggregation.

**Figure 6:**
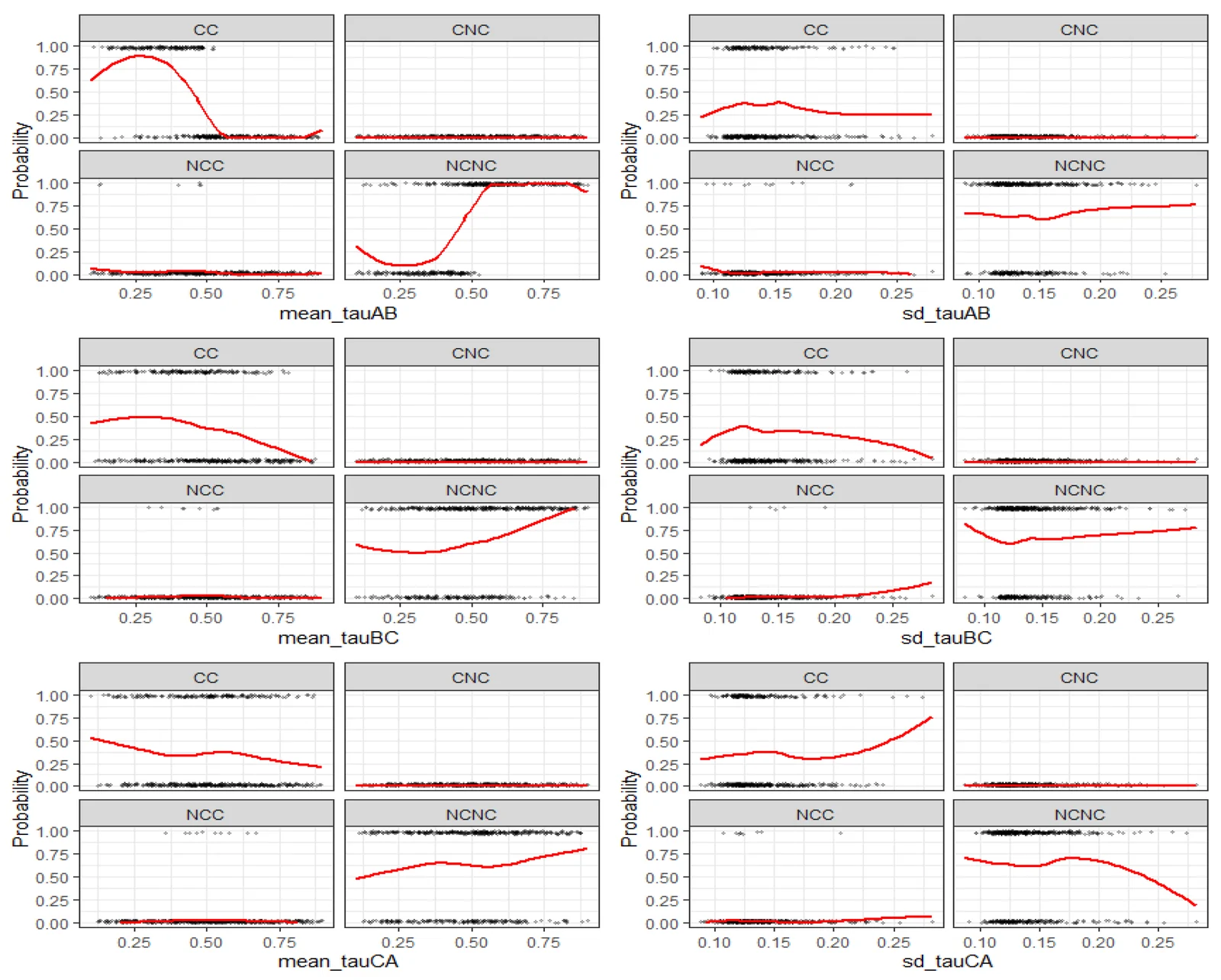
Latin hypercube probability analysis (LHPA) of micro-to-macro Condorcet’s paradox (as function of the thresholds).

The findings indicate that the evaluation of the intermediate alternative plays a central role in cycle formation. Variability in the BC threshold introduces divergent trajectories after the first comparison, while variability in the CA threshold determines whether these trajectories close into a cyclic structure. Thus, macro-level Condorcet paradoxes appear to emerge less from the initial preference relation and more from heterogeneous assessments of intermediate and subsequent comparisons (see Figure 6).

## 4. Discussion

From a broader perspective, and particularly when collective decisions are viewed as complex or even quantum-like systems, Condorcet’s paradox suggests a further limitation: individuals do not have direct access to the global consequences of their actions. While each voter expresses a locally coherent preference ordering, the aggregation process may generate emergent outcomes that cannot be inferred from any individual ranking alone. In this interpretation, social choice becomes a system in which collective properties emerge from interactions among agents and are not reducible to the intentions or knowledge of any single participant. The paradox therefore highlights not only the possibility of cyclical majorities, but also the epistemic limits that characterize collective decision-making in complex environments.

### 4.1. Contextual Freedom and Epistemic Transparency: A New Impossibility Statement in Social Choice

Following [14] and the above analysis, there is an incompatibility between contextual freedom and complete epistemic transparency in collective decision-making. In the spirit of the impossibility theorems [2,3], it identifies a structural tension between two desirable principles. On the one hand, individuals are free to adopt different, yet internally coherent, preferences across decision-making contexts. On the other hand, one may wish every individual to be able to infer the full consequences of collective decisions from their own local perspective. These objectives cannot, in general, be simultaneously achieved. Beyond a certain degree of contextuality, the collective structure acquires genuinely global features that are not reducible to any local viewpoint. As a consequence, if a society preserves the freedom of individuals to choose differently across contexts, then some information about the aggregate outcome necessarily remains inaccessible at the individual level, i.e., an individual may observe the elected alternative but cannot in general reconstruct from local information the global preference structure that made that outcome possible.

### 4.2. Non-Constant Contextuality

In the original model, the contextual coupling parameter γ is assumed to be constant. Consequently, the strength of contextual influences remains unchanged throughout the decision process. This assumption is useful for isolating the effects of contextual interactions, but it is not necessary from a dynamical perspective. A natural extension consists in allowing contextuality itself to evolve over time. Let denote $\gamma_{t}$ the contextual coupling at time t. Rather than remaining fixed, contextuality may follow an intrinsic nonlinear dynamic, where *F* is a nonlinear map. In this formulation, the probability of observing a Condorcet cycle becomes a consequence of the current level of contextuality, rather than a factor influencing it.

The Condorcet paradox therefore acts as an observable of the contextual state of the system. As contextuality changes, the prevalence of collective cycles changes accordingly. Stable contextual dynamics lead to stationary levels of Condorcet cycles, while oscillatory contextual dynamics generate periodic fluctuations in the probability of the paradox.

More interestingly, if the evolution of contextuality is governed by strongly nonlinear dynamics, such as logistic or tent-like maps, the system may undergo bifurcations and eventually enter a chaotic regime. In such cases, the frequency of Condorcet cycles exhibits irregular fluctuations despite the deterministic character of the underlying process.

This scenario has important implications for quantum-like behavior. In the original model with constant contextuality, increasing contextual interactions progressively drive the system toward a regime in which classical representability breaks down, and quantum-like descriptions become increasingly appropriate. Under chaotic contextual dynamics, however, this transition is no longer stable.

Periods of high contextuality may temporarily generate violations of classical representability and favor quantum-like collective states. Yet these intervals are continuously interrupted by periods of lower contextuality, during which classical latent-order representations become valid again. Consequently, the collective system does not converge toward a persistent quantum-like regime. Instead, it repeatedly crosses the boundary between classical and quantum-like behavior.

The resulting dynamics can be summarized as: Classical Regime ↔ Quantum-Like Regime.

Quantum-like correlations therefore become transient rather than stationary. Their appearance depends on the instantaneous level of contextuality and may be continuously created and destroyed by the underlying chaotic dynamics. This perspective suggests a possible explanation for the difficulty of observing robust quantum-like behavior in real social systems. Even when contextuality can generate quantum-like collective states, chaotic fluctuations may prevent the system from remaining in such a regime for extended periods. Observable behavior would then consist of alternating phases of classical and quantum-like organization, reflecting the intrinsic dynamics of contextuality rather than a stable probabilistic structure.

### 4.3. Non-Constant Contextuality and Global Feedback

The previous formulation assumes that Condorcet cycles are merely consequences of contextuality. Social systems, however, possess an additional property that distinguishes them from most physical systems: collective outcomes may become observable and influence subsequent individual behavior. This possibility introduces a second layer of dynamics in which the probability of Condorcet cycles feeds back into contextuality itself. A distinctive feature of social systems is that emergent global properties may become locally accessible through communication, shared knowledge, or collective awareness. Unlike most physical systems, where emergent macroscopic properties do not directly influence the microscopic constituents that generate them, social agents can observe and react to collective outcomes.

The Condorcet paradox provides a natural example of this mechanism. A cyclical collective preference may emerge from locally coherent individual rankings, and it constitutes a genuinely global property of the system. Once agents become aware of the existence of such cycles, the collective outcome ceases to be merely an emergent phenomenon and becomes part of the decision context itself.

In the original model, the contextual coupling parameter γ is assumed to be constant. Consequently, the strength of contextual effects remains unchanged throughout the decision process. Here, we relax this assumption and treat contextuality as a dynamic property that evolves in response to collective outcomes.

Let $P(\gamma_{t})$ be the probability of observing a Condorcet cycle under the contextual coupling level $\gamma_{t}$. We assume that contextuality evolves according to:

(12) $$\gamma_{t+1}=\gamma_{t}+r-\alpha \;max(\;P(\gamma_{t}\;)-\theta ,\;0),\;\;$$

where r>0 represents the natural tendency of agents to recover contextual freedom, θ is the threshold above which the paradox becomes collectively noticeable, and α measures the strength of the corrective response.

When the probability of the paradox remains below the threshold $P\left(\gamma_{t}\right)<\theta ,$ collective awareness is weak and agents experience no significant pressure toward coherence. Contextuality therefore increases according to $\gamma_{t+1}=\gamma_{t}+r.\;$ However, once the probability of Condorcet cycles exceeds the critical threshold, $P\left(\gamma_{t}\right)>\theta$, the paradox becomes socially visible. Agents then attempt to restore consistency by reducing their dependence on contextual influences. The larger the excess probability of the paradox, the stronger the reduction in contextual coupling. This creates a self-referential feedback loop: Contextuality → Condorcet Cycles → Collective Awareness → Reduction in Contextuality.

Importantly, the corrective mechanism also weakens the very signal that activated it. As contextuality decreases, the probability of observing Condorcet cycles falls. Once this probability drops below the perceptual threshold, awareness gradually disappears and agents recover contextual freedom, allowing contextuality to increase again. The system therefore alternates between two regimes: Contextual Regime ↔ Coherence Regime.

In the contextual regime, contextual influences grow and generate an increasing probability of collective inconsistency. In the coherence regime, awareness of the paradox induces corrective behavior that suppresses contextuality and restores consistency. For sufficiently strong feedback (α) and sufficiently nonlinear dependence of $P\left(\gamma_{t}\right)\;$ on contextuality, the induced map $\gamma_{t+1}={F(\gamma }_{t})\;$ may acquire a piecewise-linear structure like a tent map. In this regime, repeated transitions between contextual and coherent phases can generate persistent oscillations, switching dynamics, period-doubling bifurcations, and potentially chaotic collective behavior. In this interpretation, chaos does not emerge from contextuality alone. Rather, it arises from the continual interaction between contextuality, collective inconsistency, awareness, corrective adaptation, and the subsequent recovery of contextual freedom. The Condorcet paradox therefore becomes not only an emergent manifestation of contextual interactions, but also a dynamic regulatory mechanism governing the evolution of contextuality itself.

### 4.4. Contextuality, Feedback, and Quantum-like Behavior

The emergence of global-to-local feedback transforms the system from a purely contextual system into a reflexive system. In a purely contextual regime, correlations originate exclusively from contextual dependencies and may exhibit quantum-like features. However, when global collective properties become locally accessible, an additional causal channel appears. The collective state influences individual decisions, which in turn regenerate the collective state. This recursive mechanism introduces an additional causal channel linking collective states and individual decisions. Consequently, the system is no longer purely contextual. Collective outcomes become part of the information available to agents and therefore contribute directly to the generation of future behavior.

For instance, a third form of contextuality may arise when individuals are aware that the final decision will be determined through majority rule. In this case, the aggregation mechanism is no longer an external institution acting after preferences have been formed. Instead, it becomes part of the decision context itself. Individuals may form, revise, or strategically express their preferences while anticipating the collective consequences of their choices. Consequently, majority rule participates in the construction of preferences rather than simply aggregating them.

Under this interpretation, contextuality is neither purely cognitive nor purely institutional, but reflexive. It originates from the interaction between individual beliefs and expectations regarding the collective decision process. Preferences are formed not only in relation to the available alternatives, but also in relation to the anticipated aggregation mechanism.

The resulting framework therefore distinguishes three sources of incompatibility. In the *micro-Condorcet paradox*, incompatibility is *cognitive*, emerging from contextual interactions within individual decision makers. In the *macro-Condorcet paradox,* incompatibility is *institutional*, emerging from the constraints imposed by majority aggregation. In this third case, incompatibility is *reflexive*, emerging because agents incorporate the aggregation rule into their own preference formation process.

This consideration leads us to introduce the concept of the anticipated Condorcet paradox. In the classical macro-Condorcet paradox, collective cyclicity emerges even though individual preferences are assumed to be coherent and independently formed. However, real decision makers may be aware that the final outcome will be determined through majority rule. Under these conditions, the aggregation mechanism itself becomes part of the preference-formation process.

Individuals no longer evaluate alternatives solely according to their intrinsic preferences, but also according to their expected impact on the collective outcome. As a result, agents may rationally modify the ranking they reveal in order to increase the probability that if their most preferred alternative cannot prevail, at least their second-best alternative will. The preference expressed by the individual therefore reflects not only an ordering over alternatives, but also expectations about the aggregation process.

In this perspective, the Condorcet cycle does not arise despite rational behavior, but because of it. Collective inconsistency emerges from the strategic adaptation of rational individuals to an anticipated institutional rule. What appears as a paradox at the collective level may therefore represent a higher-order form of rationality operating under majority aggregation constraints.

From a quantum-like perspective, the anticipated Condorcet paradox corresponds to a third form of contextuality. The context is no longer generated solely by previous decisions (*cognitive contextuality*) or by the aggregation rule itself (*institutional contextuality*), but by agents’ expectations concerning the future collective outcome. The aggregation rule becomes part of the state space within which preferences are formed, creating a feedback loop between individuals and institutions.

The three paradoxes can therefore be interpreted as three distinct manifestations of contextuality. Cognitive contextuality originates within the decision maker, institutional contextuality originates within the aggregation mechanism, and reflexive contextuality originates from the anticipation of the aggregation mechanism and the resulting interaction between cognition and institutions. Although their causal origins differ, all three forms share the same structural signature: local preference relations may fail to admit a single globally consistent classical representation. Consequently, the resulting preference structures may be naturally represented within a quantum-like probabilistic framework.

### 4.5. Effect of Social Interaction

Social influence may reduce the occurrence of macro-Condorcet cycles not only by increasing preference alignment, but also by reducing the effective non-communicability among decision makers. As information, arguments, and evaluations circulate through the social network, individual preference orderings become partially correlated. The resulting collective state is therefore less fragmented, making the emergence of cyclical majorities less likely. In a quantum-like interpretation, social interaction acts as a coherence-inducing mechanism that partially suppresses contextual inconsistencies at the aggregate level. Furthermore, social interaction removes the assumption of independence among local contexts that underlies the non-signaling analogue of the Condorcet framework. Once agents can influence one another through communication, observed correlations may be explained by explicit information transfer rather than by contextual collective organization alone. Consequently, increasing social interaction tends to move the system away from the conditions under which quantum-like correlations are most naturally expected to emerge.

## 5. Conclusions

The paper shows that contextual interactions among locally coherent agents can generate collective states that are incompatible with classical latent-order representations. The disappearance of classical feasibility coincides with the emergence of Condorcet cycles, increased correlations, and a region of maximal configurational complexity.

Most importantly, the breakdown of classical representability does not lead to a loss of structure. Instead, the collective system remains highly organized and continues to admit an accurate quantum-like representation. Moreover, our results show that the explanatory adequacy of the quantum-like framework increases with contextuality. While quantum-like models provide a satisfactory description across the entire parameter space, their predictive accuracy improves after the critical threshold at which classical representability breaks down. This suggests that increasing contextuality progressively favors quantum-like probabilistic structures over classical latent-order representations.

More broadly, the findings indicate that quantum-like descriptions need not be assumed a priori [14], as usually done in quantum decision-making [15,16,17,18,19]. Rather, they may emerge naturally as higher-level representations of collective systems whose contextual organization exceeds the limits of classical probabilistic frameworks. Even more importantly, this framework eliminates the need to assume cognitive superposition states prior to decision-making. The relevant contexts emerge through the presentation of alternatives themselves, and in the absence of a global representation encompassing all possible options, there is no theoretical requirement to invoke superposition as a fundamental feature of the cognitive process [20,21,22] employed in quantum decision-making [23,24,25]. While quantum effects in physics are associated with microscopic scales governed by Planck’s constant, where superposition is disrupted through measurement, in the social macro-world, quantum-like effects arise from contextuality. Here, emergence is not rooted in physical superposition, but in the context-dependent construction of meaning and choice [14]. Quantum-like effects arise at the collective level from this global contextual emergence and can be effectively modeled through a quantum probabilistic framework, also showing the typical violations of Bell’s inequalities in the macroworld [26,27,28].

## Figures and Tables

**Table 1:** Contextuality, local, and global coherence of preferences: a taxonomy.

		Global Transitivity	Global Non-Transitivity
**No contextuality**	**Local transitivity**	*Classical coherence* Both individual and collective preferences admit a consistent ordering.	*Macro-Condorcet’s paradox* Locally coherent preferences generate an intransitive collective ordering.
**Contextuality**	**Local non-transitivity** *(Micro-Condorcet’s paradox)* Impossibility of reproducing all the contextual preferences adopting a coherent preference system	*Incoherence cancellation* Individual preference cycles disappear through aggregation, yielding a coherent collective ranking.	*Reinforced incoherence* Non-transitivity is present at both the individual and collective levels.

## Data Availability

The R code (Version R 4.3.2.) used to generate and analyze the simulations presented in this study is available from the corresponding author upon reasonable request.

## Abbreviation

The following abbreviation is used in this manuscript:

RMSE	Root mean squared error
